# Casting Aluminum

**Published:** 1870-07

**Authors:** 


### CASTING ALUMINUM.

We recently witnessed some experiments in casting aluminum plates for artificial teeth, resulting in all that could have been anticipated or desired. The plates are very perfect, thin and light; and so little shrinkage as not to interfere with their adaptation, to any appreciable degree, at least. The process we could wish more simple, and very probably it will be made so. There are three members of the profession in this and an adjoining State; each of whom has devised a plan, and carried out the process for casting aluminum for dental purposes, and others too, for that matter, each independent of the other. Their methods, of course, require some skill for their carrying out; and it is not to be regretted that it is so; for our profession, just now, very much needs to be replenished with men who have the ability to become both skillful and scientific. There are men in our profession who are exceedingly fertile in skillful resources, but they are few, very few; the great mass being entirely content to be mere imitators of what others have done. We are not aware that the casting of aluminum for light, thin articles has been made a success, outside of the dental profession. There will be an opportunity, soon, for the profession to obtain instructions in this work. T.

